# Editorial: Disease and pest resistance in legume crops

**DOI:** 10.3389/fpls.2023.1166387

**Published:** 2023-03-13

**Authors:** Sukhjiwan Kaur, Maria Celeste Gonçalves-Vidigal, Jennifer Davidson, Kirankumar S. Mysore, Abhay K. Pandey

**Affiliations:** ^1^ Agriculture Victoria, AgriBio, Centre for AgriBioscience, Melbourne, VIC, Australia; ^2^ Departamento de Agronomia, Universidade Estadual de Maringá-UEM, Maringá, Brazil; ^3^ School of Agriculture, Food and Wine, Waite Research Institute, The University of Adelaide, Adelaide, SA, Australia; ^4^ Department of Biochemistry and Molecular Biology, Oklahoma State University, Stillwater, OK, United States; ^5^ Institute for Agricultural Biosciences, Oklahoma State University, Ardmore, OK, United States; ^6^ Department of Mycology & Microbiology, Tea Research Association, North Bengal Regional R & D Center, Jalpaiguri, West Bengal, India

**Keywords:** disease resistance, pest resistance, genomic selection, crop management strategies, germplasm characterization, crop wild relatives

In the past decade, food legumes that show potential increase in production are chickpea, mungbean, blackgram, soybean, lentil, chickpea and faba bean. Besides that, common bean keeps being a crucial staple food. However, production of these food legumes is significantly hampered by both biotic and abiotic stresses (Nair et al., 2019; Boufleur et al., 2021). Major diseases of legumes include powdery and downy mildews, *Botrytis* grey molds, root rots, Ascochyta blights, anthracnoses, rusts, wilts, bacterial blights and mosaic diseases. In addition, damages caused by nematodes, parasitic weeds, and chewing/sap-sucking insects like pod borers/whitefly add to this long list of constraints for legume production.

Plant breeding programs aim to develop pest and disease-resistant varieties. To accurately characterize germplasm, it is crucial to understand how genetics and host-pathogen/pest interactions work (Rubiales et al., 2015). In addition, identifying novel alleles, inter-crossing and backcrossing strategies can enrich breeding germplasm through the use of wild relatives of crops. Alternatively, selection for disease and pest resistance is through high throughput field phenotyping. Genomic technologies can enable the identification and characterization of resistance genes and the functional characterization of their products (Mukankusi et al., 2019).

The objectives of the Research Topic on *Disease and Pest Resistance in Legume Crops* were to compile advances in research towards managing diseases and pests through various omics approaches, mechanism underlying host-pathogen interactions, germplasm characterization using modern genomics and phenomics tools, and implementation of novel approaches for disease and pest resistance in legumes that can be used in crop improvement. The topic received a tremendous response from scholars, with nine accepted articles contributed by 59 authors from worldwide.

Martins et al. investigated the genetic architecture of grass pea resistance to *Uromyces pisi* through a genome-wide association approach. The authors reported six single-nucleotide polymorphism (SNP) markers linked with disease severity, signifying that partial resistance is oligogenic, located on chromosomes 4 and 6. After mapping with pea reference genome, (Mukankusi et al., 2019) proposed 19 candidate genes encoding for leucine-rich repeat, NB-ARC domain, and TGA transcription factor family, among others, which might help in understanding the molecular mechanisms of quantitative resistance to rust in grass pea.

Yang et al. reported that cinnamic acid enhances wilt in faba bean (*Vicia faba* L.) caused by *Fusarium oxysporum* f. sp. *fabae* by increasing activity of cell wall degrading enzymes and content of lignin in the stem produced by pathogen. To mitigate this problem, they found that intercropping of faba bean with wheat reduced the occurrence of wilt by decreasing the activity of cell wall degrading enzymes.

Joshi et al. revealed field pea (*Pisum sativum* L.) resistance against Ascochyta blight, caused by *Peyronellaea pinodes* and *Didymella pinodella.* Their study reports varieties with high levels of resistance against both pathogens and susceptible variety to be used as a susceptible check in disease screening program. They also found that in resistant genotypes, accumulation of hydrogen peroxide was lower compared to susceptible genotype.

*Macrophomina phaseolina* (Tassi) Goidanich causes dry root rot and blight diseases in many legumes and ashy stem blight (ASB) in common bean. In this context, Viteri et al. identified major quantitative traits loci (QTLs) and SNP markers associated with ashy stem blight resistance. Two SNPs, Chr03_39824257 and Chr03_39824268 were identified as the strongest markers associated with resistance to this disease and the drought sensitive gene *Phvul.003G175900* was recognized as one candidate for ASB resistance in the recombinant inbred lines (RIL).

Another study on genetic mapping and inheritance of resistance of common bean against anthracnose was carried out by Gomes-Messias et al.. Their findings revealed that anthracnose resistance in BRSMG Realce (an Andean bean [*Phaseolus vulgaris* L.] cultivar) is controlled by a major resistance gene, i.e., *Co-Realce* located on chromosome Pv04, flanked by SNP markers, snp1327 and snp12782 at a distance of 4.48 cM apart each other. Thus, a selection efficiency of 99.2% makes these SNPs suitable for marker-assisted selection (MAS).

Ferreira et al. reported two transgenic whitefly-tolerant common bean lines with an intron-hairpin construct to induce post-transcriptional gene silencing against *Bemisia tabaci vATPase* (*Bt*-*vATPase*) gene, with stable expression of siRNA. When compared to non-transgenic controls, insects fed on the transgenic line Bt-22.5 expressed 50% less *Bt*-*vATPase*. Whitefly-tolerant transgenic elite common bean cultivars can be developed contributing to the management of whitefly and viral diseases in common bean.

Taboada et al. review of literature revealed that white mold incited by *Sclerotinia sclerotiorum*, angular leaf spot by *Pseudocercospora griseola*, and web blight and root rot by *Rhizoctonia solani* were the major fungal diseases threatening common bean production in Argentina. Morpho-molecular features of about 200 isolates of these pathogens are discussed in this review along with screening of common bean genotypes under controlled and field conditions.

Parihar et al. reviewed genomics breeding strategies for major biotic stresses in Pea (*Pisum sativum* L.). Several QTLs and genetic markers associated with genes controlling resistance to pea diseases available for marker-assisted breeding are summarised in this review. In the long run, a judicious combination of conventional and cutting-edge omics-based breeding strategies will enhance genetic gain and optimize the development of biotic stress-resistant cultivars in order to sustain pea production in changing climates.

Roy et al. published a systematic review on breeding approaches for disease resistance in lentil (*Lens culinaris* Medik). They summarised the major genetic resources of lentil, disease screening methods and molecular markers associated with disease resistance that can be used in MAS program after further genetic validation in different genetic backgrounds. Roy et al. also focuses on mutation breeding, and recent interventions in omics technologies including CRISPR/Cas9 technology for improving disease resistance in lentil with advantages and limitations.

Research contributions to this Research Topic highlight the multiple dimensions of disease and pest resistance in legumes. In addition, the topic also covers disease screening techniques, the role of conventional and omics-based breeding approaches in improving yield limitation caused by major pests and diseases, and progress toward making legume varieties more resilient to disease or pest outbreaks under the shadow of climate change.

## Author contributions

All authors contributed to the review of manuscripts and preparation of this editorial. All authors contributed to the article and approved the submitted version.
